# The Ether Patent

**Published:** 1861-01

**Authors:** 


THE ETHER PATENT.—The Hon. P. F. Thomas, Commissioner of Patents at Washington, deserves the thanks of the medical profession and the public at large, for refusing to renew the patent issued to Dr. Morton and Dr. Jackson, fourteen years ago, for the exclusive employment of ether to induce anæsthesia in surgical operations. The patent expired on the twelfth of November, 1860, and Dr. Morton, some months since, applied for an extension of the patent for a period of seven years. Dr. Jackson, however, would not assign to Dr. Morton the right of extension, and remonstrated against it. On this account the renewal was not granted, and the result has been that the would-be patentee has succeeded in preserving his name from an amount of odium from which he could never have recovered. Such a boon should be as free as the air of heaven.

It is strange, passing strange, that Dr. Morton, after having achieved so wonderful a discovery, should have been so insensible to his fame as to seek for a patent right. Poverty is a thousand times preferable, under such circumstances, to the most inexhaustible riches extorted from the groans of the people. After indorsing Dr. Morton's claims, as we were induced to do last winter, we deeply regret that he should have permitted himself to go again before Congress. We sympathize with him in his poverty, brought on, as he alleges, by his attempts to introduce the use of ether, as an anæsthetic agent, to the notice of the profession and the public, and we think it is a burning shame upon our country and upon the age, that our National Legislature has not made him a liberal compensation for his great and inestimable services. If the labors of Jenner in the cause of vaccination deserved the recognition of the British Parliament, surely Dr. Morton is entitled to the gratitude of his countrymen for the sacrifices which he has made to furnish them with a safe means of preventing pain in surgical operations and in the throes of parturition.

